# Multivariable Time Series Prediction for the Icing Process on Overhead Power Transmission Line

**DOI:** 10.1155/2014/256815

**Published:** 2014-07-17

**Authors:** Peng Li, Na Zhao, Donghua Zhou, Min Cao, Jingjie Li, Xinling Shi

**Affiliations:** ^1^Department of Electronic Engineering, Yunnan University, Kunming 650091, China; ^2^Department of Automation, Tsinghua University, Beijing 100084, China; ^3^Yunnan Electric Power Research Institute, China Southern Power Grid Corp., Kunming 650217, China

## Abstract

The design of monitoring and predictive alarm systems is necessary for successful overhead power transmission line icing. Given the characteristics of complexity, nonlinearity, and fitfulness in the line icing process, a model based on a multivariable time series is presented here to predict the icing load of a transmission line. In this model, the time effects of micrometeorology parameters for the icing process have been analyzed. The phase-space reconstruction theory and machine learning method were then applied to establish the prediction model, which fully utilized the history of multivariable time series data in local monitoring systems to represent the mapping relationship between icing load and micrometeorology factors. Relevant to the characteristic of fitfulness in line icing, the simulations were carried out during the same icing process or different process to test the model's prediction precision and robustness. According to the simulation results for the Tao-Luo-Xiong Transmission Line, this model demonstrates a good accuracy of prediction in different process, if the prediction length is less than two hours, and would be helpful for power grid departments when deciding to take action in advance to address potential icing disasters.

## 1. Introduction

With rapid societal and economic development, human beings have come to rely more and more on all kinds of energy sources, but especially on electric power. Consequently, reliability, maintainability, and safety requirements have become more and more important for electric power plants and grids. In recent years, global greenhouse effects have resulted in atrocious weather events, including freezes, windstorms, severe thunderstorms, fog, floods, and mudslides. These events have threatened the safety of power grid systems. In the Southern of China, most notably in Yunnan, Guizhou, Sichuan, Chongqing, and Hunan, frozen disasters occur frequently in winter; overhead power lines pass through high altitude and low latitude areas across complex landscapes and through changing climates, such as the Qinling Mountains, the Hengduan Mountains, and the Wumeng Mountains. During the winter months, these are areas where cold air from the North meets warm, humid air from Southern, and under special micrometeorology conditions, water will ice across the surface of overhead power lines, eventually causing problems, such as tower collapse, line breakage, flashover of insulators, or conductor gallop. In early 2008, in Central Southern and Southwest China, an ice storm caused more than 120,000 towers to collapse, downed more than 7,000 electrical lines, and shut down 859 substations, according to statistics from the Southern Power Grid Corp. of China [1]. These accidents resulted in significant financial losses to domestic and industrial electrical power users.

It is always preferable to take preventative actions instead reacting to events after an accident has already happened. Line icing has caused a lot of problems to grid systems in the past; obviously, if it is possible to monitor and predict potential icing threats to transmission lines, timely alarms could improve the power grid's reliability.

The models and methods for monitoring and predicting icing vary. In the area of research focused on monitoring the icing process online, Savadjiev and Farzaneh [2] presented the IRM (icing rate meter) method, which indirectly describes the ice's rate of increase through the use of an instrument installed near the transmission line. This method is convenient but imprecise when measuring the load of line icing. Huang and Sun [3] and Yang et al. [4] presented a mechanical model that has advantages in timely and exact estimates for the load of lines icing, but the instruments for sampling the force data are expensive. This method is unable to forecast the icing status; it can only display the real-time status of the icing process. The Glouba model [5] and the edge of image extraction model [6] succeed in practice; they can estimate the icing load at a low cost, but their precision is less than the mechanical model and they are also unable to forecast icing status.

**Figure 1:**
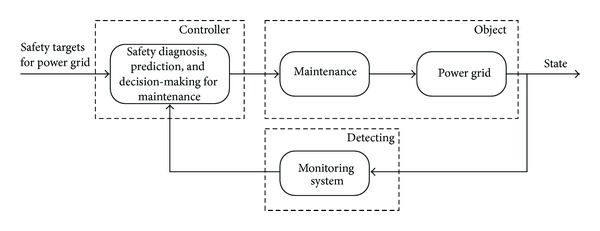
Maintenance based on safety status prediction.

As shown in Figure 1, it is obvious that decision-making based on a predictive status for maintenance is better than one based on a current status, because the predictive control has the advantage of reducing unsafe factors in advance, compared to a simple feedback control loop. Therefore, if the transmission line's icing status has been predicted and unsafe parts of it have been assessed, the departments of power grid maintenance can actively optimize a schedule to deice the lines.

Consequently, it is imperative to find methods to predict the icing threat of power grid using meteorology information, forecasted by the weather bureau.

In the area of research focused on predicting the icing process, an experiment model [7] and a statistical analysis model [8] based on micrometeorology parameters predicted icing loads using certain micrometeorology parameters, such as the environmental temperature, humidity, wind velocity and direction, rainfall, and snowfall. The Makkonen model [9] is based on a thermodynamic mechanism, but its variables, such as the content of liquid state water and a distributing dripping parameter, can be obtained in a laboratory but are difficult to measure at local monitoring points along a transmission line; so it is not generally suitable for the icing process.

Even though the mathematical models above have been obtained, they are founded in special conditions, either by statistical analysis methods or thermodynamic mechanisms, and are not robust enough to withstand if the environment changes, because the micrometeorology conditions are different in each instance when lines ice over, for example, in a valley versus along a hillside. Furthermore, because of the complexity and nonlinearity of the icing models, it is difficult to acquire all of them to predict icing process in different locations, because the mapping relationship changes between the independent variables and dependent variables.

In the northeast area of Yunnan Province in China, icing disasters happen frequently [10]. Plentiful advanced force measurements and microweather stations have been installed for the online monitoring of power grids, and the real-time icing load status and micrometeorology parameters are transmitted concurrently to a surveillance center by wired and wireless sensor networks. But as a result of the complexity in microlandforms and micrometeorology in this area, it is difficult to find a specific mathematics model to predict the icing process in different transmission lines.

On the one hand, the cost of an online monitoring system is expensive to establish and maintain, and they are unable to predict disasters in advance. On the other hand, the mass history of mechanical data and meteorology data from online monitoring system has been recorded continuously; this history reflects the real mapping relationship between disasters and factors.

Consequently, it is significant to mine helpful information from mass history data using intelligent algorithms [11–14]. By these means, we not only can take full advantage of the mass data from expensive instruments, but can also determine the prediction models for icing threats. McComber et al. [15], Larouche et al. [16], and others [17] presented a model based on an artificial neural network to predict the icing process using history data, but those models do not take into consideration the relationship between icing loads and the time effects of micrometeorology. Li et al. [18] and Li et al. [19] established a model describing the time effects of micrometeorology on line icing based on a time series analysis and machine learning methods but did not define how best to find the multivariable time series.

In this paper, we have presented a model based on a multivariable time series to predict the icing load of a power transmission line, which utilizes the history meteorology data from the icing monitoring system of China Southern Power Grid Corp. (CSPGC) and takes into account the time effects of meteorology factors using the phase-space reconstruction theory to establish it. According to the simulation results, this model accurately predicted the icing load in the Tao-Luo-Xiong Transmission Line and would prove helpful for power grid departments in their efforts to take action against icing disasters.

## 2. Prediction Model Based on Multivariable Time Series

### 2.1. Time Effects Analysis of Influence Factors in the Icing Process

**Figure 2:**
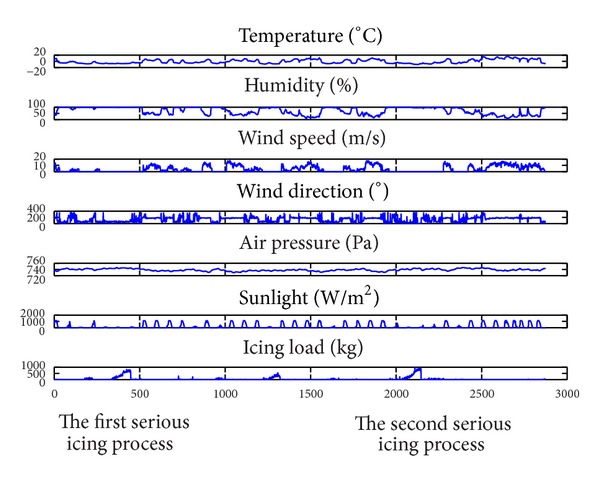
Meteorology data and icing load curves I (2009.12.14–2010.1.25).

Due to the huge damage of freezes disaster in January 2008, CSPGC has built the icing monitoring systems in some important overhead transmission lines in the northeast area of Yunnan Province. The micrometeorology and force data are collected by field sensors and synchronously transmitted to monitoring center through GSM (global system for mobile) communication system.  Figure 2 shows three icing processes, two of which are serious, in the Tao-Luo-Xiong Transmission Line from December 14th of 2009 to January 25th of 2010. There is an obvious relationship between the icing load (weight) and influence factors, such as the environment temperature, humidity, wind speed, wind direction, air pressure, and sunlight intensity.

Following are the approximate increasing conditions for the icing process in this line:

1. humidity is above 90%;
2. wind direction is about 70°;
3. wind speed is close to 0 m/s;
4. air pressure is above 735 Pa;
5. sunlight is minimal during the day;
6. temperature is below 0°C.

If those conditions continue and temperature continues to drop, the ice rain will continue for a significant amount of time; consequently, the degree of icing will become more serious.

As the Makkonen model [9, 20] describes, the icing accretion process is formulated as follows:

(1) $$\begin{array}{c} dq_{W}\left(t\right)=2R\left(t\right)\alpha_{1}\left(t\right)\alpha_{2}\left(t\right)\alpha_{3}\left(t\right)H\left(t\right)W_{S}\left(t\right)dt, \end{array}$$

where *R*(*t*) is the equivalent radius of the icing line, which restricts the surface area of the icing line that the ice rain can cling to. *α*_1_(*t*) is the collision efficiency, which is determined by forces of aerodynamic drag and inertia for water droplets. *α*_2_(*t*) is the sticking efficiency, which denotes the efficiency with which a water droplet hits an iced-over surface such that it rapidly freezes and does not bounce. *α*_3_(*t*) is the accretion efficiency. During the dry-growth icing process, *α*_3_(*t*) = 1; during wet-growth icing, the freezing rate is controlled by the rate at which the latent heat is released during the freezing process, 0 < *α*_3_(*t*) < 1. *H*(*t*) is the environment humidity and *W*_*S*_(*t*) is the wind speed.

Assuming *R*_0_ is the radius of the line without icing, we can obtain the following estimation formula:

(2) qW(t)=∫t0t2R(τ)α1(τ)α2(τ)α3(τ)H(τ)WS(τ)dτ +q0,R(t)=(R0−(4qW(t)/9.8πγ)+R02)2+R0,

where *q*_0_ is the weight of ice per unit length for the transmission line at initial time *t*_0_ and *γ* is the average density of the ice.

Using formula (2), (3) is deduced

(3) qW′(t)=(3R0−4qW(t)9.8πγ+R02) ×α1(t)α2(t)α3(t)H(t)WS(t).

If defining *u*(*t*) = *α*_1_(*t*)*α*_2_(*t*)*α*_3_(*t*)*H*(*t*)*W*_*S*_(*t*), *T* as the sampling period and assuming *T* = 1, then the state equation of the icing process is shown as the following formula:

(4) $$\begin{array}{c} q_{W}^{\prime }\left(k\right)=f\left(q_{W}\left(k-1\right),u\left(k-1\right)\right)\;k=1,2,\ldots ,n. \end{array}$$

As formula (4) shows, the state of the icing process is driven by time series *u*(*k*), and *u*(*k*) is related to micrometeorology factors, including environment temperature, humidity, wind speed, wind direction, air pressure, and sunlight intensity [19]; as formula (5) shows:

(5) $$\begin{array}{c} u\left(t\right)=\Gamma \left(T_{P}\left(t\right),H\left(t\right),W_{S}\left(t\right),W_{D}\left(t\right),A_{P}\left(t\right),S_{I}\left(t\right)\right). \end{array}$$

It is also possible to describe formula (4) using formula (6) as a discrete state equation:

(6) qW(k)=g(qW(k−1),TP(k−1),H(k−1),WS(k−1),WD(k−1),AP(k−1),SI(k−1)),

where the variables are defined as follows: *q*_*W*_: icing load (weight) of transmission line; *T*_*P*_: environment temperature; *H*: environment humidity; *W*_*S*_: wind speed; *W*_*D*_: wind direction (clockwise angle departure from north); *A*_*P*_: air pressure; *S*_*I*_: sunlight intensity; *k*: sampling time; *g*(∗): mapping function.

The process of line icing can be regarded as the icing load at time *k*, which is decided by icing load and micrometeorology factors at time *k* − 1, that is, a multivariate time series process.

### 2.2. Icing Load Time Series Model Based on Phase-Space Reconstruction and Machine Learning

But as shown in Figure 2, it is evident that the process of transmission line icing is fitful and complex; icing or melting often happens alternately over several months, and the increasing or decreasing of the icing process is nonlinear and nonstationary. Thus, it is difficult to find the mapping function *g*(∗) in (6).

A chaos time series prediction theory based on phase-space reconstruction has been widely applied in prediction for multivariable and nonlinear time series [21, 22]. In this paper, it is the theoretical foundation for modeling the icing process.

#### 2.2.1. Phase-Space Reconstruction of Multivarious Time Serious for Icing Process

According to Takens' embedding theorem [23] for chaos time series modeling, a time series can be defined as {*x*_*i*_}_*i*=1_^*l*^, where *l* is the length of the time series, which can be reconstructed from a *D*-dimensional phase space, described by the following formula:

(7) $$\begin{array}{c} x_{i}\left(t\right)={\left[x_{i}\left(t\right),x_{i}\left(t-\tau \right),\ldots ,x_{i}\left(t-\left(m-1\right)\tau \right)\right]}^{T}, \end{array}$$

where *m* is known as the embedding dimension of reconstructed phase space and *τ* is the delay constant. Because the power transmission line icing is a multivariate time series described by formula (6), according to the phase-space reconstruction model shown as formula (7), the time series prediction model of power transmission line icing can be found as the following formula:

(8) V(n)=[qW(n),qW(n−τ1),…,qW(n−(d1−1)τ1),TP(n),TP(n−τ2),…,TP(n−(d2−1)τ2),H(n),H(n−τ3),…,H(n−(d3−1)τ3),WS(n),WS(n−τ4),…,WS(n−(d4−1)τ4),WD(n),WD(n−τ5),…,WD(n−(d5−1)τ5),AP(n),AP(n−τ6),…,AP(n−(d6−1)τ6),SL(n),SL(n−τ7),…,SL(n−(d7−1)τ7)]T,

where *n* is the total number of sampling for icing process time series and *τ*_*i*_ and *d*_*i*_ are the time delay and embedding dimension for each variable, respectively, *i* = 1,2,…, 7. Based on Takens' embedding theorem [23], if *d* = ∑_*i*=1_^7^ *d*_*i*_ is large enough, and 1 ≤ *k* ≤ *a*, *a* is the most step length for prediction, and the mapping function Φ^(*d*)^ is available:

(9) $$\begin{array}{c} V\left(n+k\right)=\Phi^{\left(d\right)}\left(V\left(n\right)\right). \end{array}$$

Formula (9) is equal to (10) as follows:

(10) qW(n+k)=Φ1(V(n))TP(n+k)=Φ2(V(n))⋮SL(n+k)=Φ7(V(n)).

Therefore, the mapping for *q*_*W*_(*n* + *k*) and *V*(*n*) can be described as the following formula:

(11) $$\begin{array}{c} q_{W}\left(n+k\right)=\Phi_{1}\left(V\left(n\right)\right). \end{array}$$

As Takens' embedding principle based on chaos theory indicates, selecting *τ*_*i*_ and *d*_*i*_ is important for (8), because they decide the degree of approximation for the phase space and the size of the attractor. In this paper, the autocorrelation algorithm [24] was applied to estimate the delay time *τ*_*i*_, and the saturated correlation dimension algorithm (G-P algorithm) [25] was used to select the embedding dimension *d*_*i*_.

However, if there is no noise in this time series then Takens' embedding principle [23] figures that an unoptimizable *τ*_*i*_ is not influential in its performance for phase space to describe the dynamic characteristics of a complex process. So owing to decreasing complexity of computing, we confirm *τ*_1_ = 2 and *τ*_*i*_ = 0  (*i* ≠ 1).

Values of delay time *τ*_*i*_ and embedding dimension *d*_*i*_ are shown in Table 1.

The mapping between *q*_*W*_(*n* + *k*) and *V*(*n*) is obtained as the following formula:

(12) qW(n+k)=Φ1(qW(n),qW(n−2),qW(n−4),…,qW(n−10),TP(n),H(n),WS(n),WD(n),AP(n),SL(n)).

Function Φ_1_(·) is the optimization mapping, which can be obtained using the following formula:

(13) $$\begin{array}{c} \min J=\overset{N}{\underset{n=1}{\sum }}{\left[q_{W}\left(n+k\right)-{\hat{\Phi }}_{1}\left(V\left(n\right)\right)\right]}^{2}, \end{array}$$

where ${\hat{\Phi }}_{1}(\cdot )$ is the optimal estimate for Φ_1_(·).

#### 2.2.2. Optimization Mapping Modeling of Icing Process Based on Machine Learning

**Figure 3:**
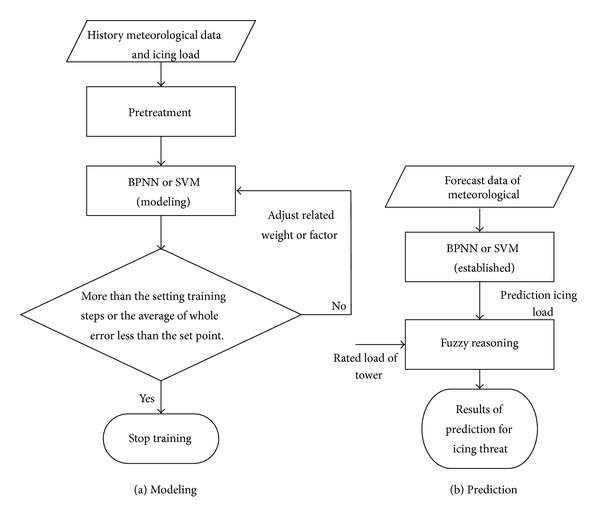
The process of modeling and prediction.

As Φ_1_(·) is multivariate and nonlinear, we can obtain it using BPNN (back propagation neural network) [26–28], SVM (support vector machine) [29, 30], and other data-driven methods. Here ${\hat{\Phi }}_{1}(\cdot )$ is founded by BPNN; it is a machine learning process, as shown in Figure 3. After training, that is also the prediction model of the icing load time series.

As formula (13) shows, the output of BPNN is the result of a prediction at time *n*, which denotes the value of icing load at time *n* + *k*. *T*_*P*_(*n*), *H*(*n*), *W*_*S*_(*n*), *W*_*D*_(*n*), *A*_*P*_(*n*), and *S*_*L*_(*n*) denote the meteorology factors, such as temperature, humidity, wind speed, wind direction, and sunlight, which impact the process of icing at time *n*.

*q*_*W*_(*n*), *q*_*W*_(*n* − 2), *q*_*W*_(*n* − 4), *q*_*W*_(*n* − 6), *q*_*W*_(*n* − 8), and *q*_*W*_(*n* − 10) are the history icing loads at time *n*, *n* − 2, *n* − 4, *n* − 6, *n* − 8, and *n* − 10, which are achieved by the mechanical model [3, 4].

In this method, given the ability to approximate any nonlinear mapping using learning in BPNN, the function ${\hat{\Phi }}_{1}(\cdot )$ was found using the model in Figure 3(a). After training the BBPN, the prediction model could be applied as shown in Figure 3(b). Determining the icing prediction values allows the relevant departments to assess the threat of inclement weather to the power grid and take some deicing or maintenance actions in advance.

## 3. Simulation and Analysis

Micrometeorology data, including environment temperature, humidity, wind velocity, wind direction, sunlight intensity, and air pressure, in addition to the insulator's mechanical data, such as pull force, wind angle, and lean angle, were acquired from an online monitoring system tracking the Tao-Luo-Xiong Transmission Line, in the northwest area of Yunnan Province, China.

The data were collected from a variety of sensors and recorded every 20 minutes from November 2009 to January 2010, as shown in Figure 2, which shows the two most serious icing processes.

For the purposes of this paper, in testing the model's precision and robustness, the simulations were carried out during the same icing process or different process.

However, the prediction model in this paper has been based on phase-space reconstruction; it is a multivariable time series prediction model, which considers multidimensional meteorology factors. If we take the icing process as a single variable time series and predict it using ARIMA (autoregressive integrated moving average) model [22], the differences between the two icing process prediction methods have also been analyzed in this paper.

As Figure 3(a) shows, the first step for finding the icing threat prediction model based on data-driven methods is to pretreat the data. Because the model is based on methods including BPNN, SVM [29], and other machine learning algorithms, the quality of data determines the precision of the model. Data pretreatment can reduce uncertain effects for modeling through the use of a filter or smoothing methods. Given that formula (12) was found without noise, data pretreatment is necessary for a prediction model based on phase-space reconstruction.

### 3.1. Data Pretreatment

As shown in Figure 2, wind speed, wind direction, sunlight intensity, and icing loads are unstable when comparing temperature, humidity, and air pressure. That can result in some disadvantages for modeling as described in the following, in which case we only performed pretreatment for those four variations.

**Figure 4:**
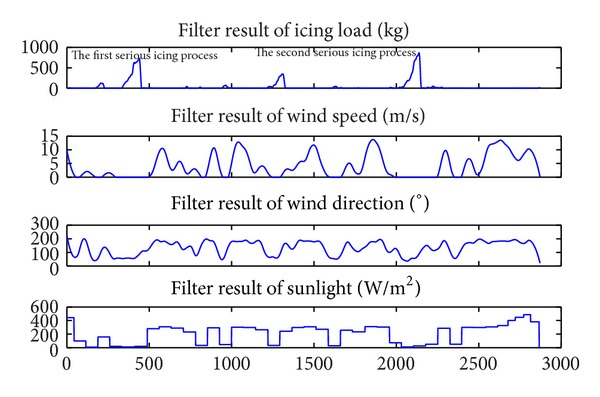
Filter results of *q*_*W*_, *W*_*P*_, *W*_*D*_, and *S*_*L*_.

(1) The sunlight intensity changes periodically. During the daytime, there is an obvious difference when the weather is clear or raining, but at night regardless whether the weather is clear or raining, the sunlight intensity is the same, equaling zero, which impacts the learning of BPNN. Having been filtered using the wavelets algorithm [31], the curve of sunlight is displayed in Figure 4. It holds certain relativity with the icing load: the icing begins and increases when the sunlight intensity is close to zero.

(2) Owing to uncertain shelling during the icing process, there exist some noise and outliers in the value of the icing load. These are determined by the shape and distribution of ice along the transmission line and not only by meteorology factors. The precision would be influenced if training with those noise and outliers. In Figure 4, the icing load has been smoothed by using weighted linear least squares model [32].

(3) As shown in the analysis in Section 2.1, the icing begins and rises when the wind direction is about 70° and wind speed is close to zero. Although wind speed and wind direction have some stability over a limited period, they change with the air current. This also impacts the training for the model. In Figure 4, the wind direction and speed have also been smoothed using the weighted linear least squares model [31].

### 3.2. Icing Load Prediction in Same Icing Process

In this case, we predicted the icing load in the same process, which means the simulation data for training and for prediction exist in the same icing process. In this icing process, as shown in Figure 2, the number of data is 230; from December 17th to 20th in 2009, the frontal 192 data were used as the training input, not including the shelling (melting) phase. The modeling flow chart of the BPNN is shown in Figure 3(a); the maximal setting of training steps is 1,000, the error value of set point is 0.01, and the earning rate parameter is 0.1.

**Figure 5:**
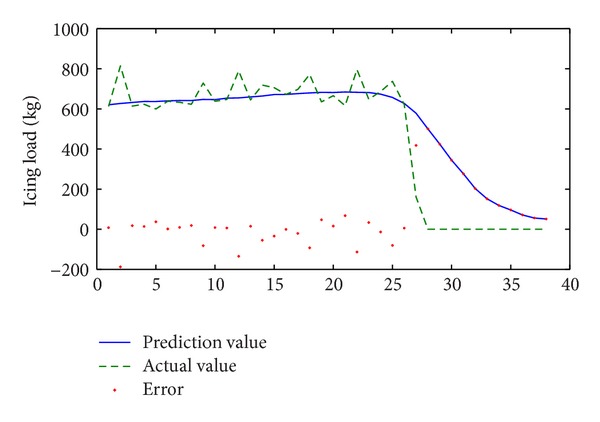
Results of prediction in the same icing process (*k* = 1) for the first serious icing process (2009.12.17–2009.12.20).

After finding the model, the remainder data were availed to prediction. As a result we achieved the prediction results as Figure 5 when the prediction length *k* is 1.

In Figure 5, the trend of change for the icing process has been predicted accurately; the error variance of prediction is 108.6 kg, which is about 14% of max icing load in this process.

If prediction length *k* increases, the prediction error variances will rise rapidly as Table 2 shows. But if *k* ≤ 4, the error variance will be less than 30% of the max icing load. This is available prediction information for power grid department to use against icing disasters.

If we do predict using the ARIMA model, it is obvious that the error variances will be greater than in the model we have shown in this paper. The results can be found in Table 2.

**Figure 6:**
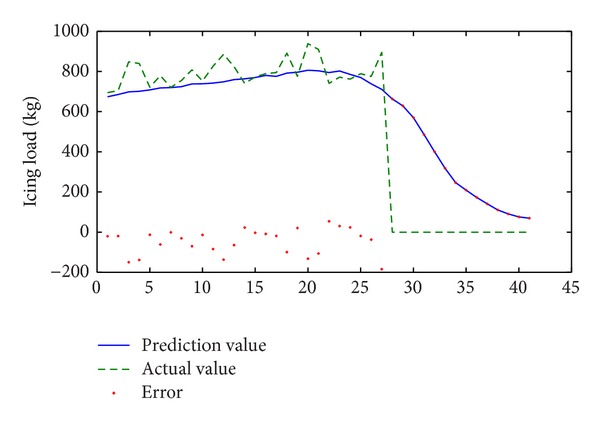
Results of prediction in same icing process (*k* = 1) for the second serious icing process (1/10/2010~1/12/2010).

Similarly, if we predict the second serious icing process as Figure 2 shows, where the number of training data is 109 from January 10th to 12th in 2010, the number of prediction data is 41 on January 12th in 2010. Results are shown in Figure 6 and Table 3.

### 3.3. Icing Load Prediction in Different Icing Process

As shown in Figure 2, we have selected the full process of the first serious icing as the training input data, which is also shown in Figure 4. The training process involves three phases, including inexistence, rising, and shelling (melting). Unlike Section 3.2, the shelling phase is used to train the BPNN. The number of training data is 310 from December 17th to 20th in 2009, the maximal setting steps of training are 1000, the error value of set point is 0.01, and the earning rate parameter is 0.1.

**Figure 7:**
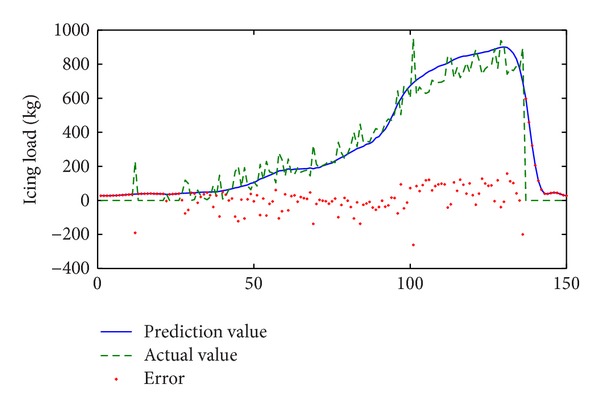
Results of prediction in different icing process (*k* = 1).

The second serious icing process is used to make predictions. The number of predictions is 150 from January 10th to 12th in 2010, which is shown in Figure 2. The results of simulation are as shown in Figure 7.

In Figure 7, the trend of change for the icing process is predicted more accurately, because the error in the shelling phase is less than found in Figure 6. The error variance of prediction is 71.2 kg, which is about 10% of max icing load in this process.

This is similar to Section 3.2 in that the prediction error variances will rise rapidly if prediction length *k* increases, as shown in Table 4. If *k* ≥ 5, the error variance will be more than 30% of the max icing load.

If we perform a prediction using ARIMA model, it is evident that the error variances are greater than found in the model with the multivariable time series. On the other hand, the error variances that were predicted in the different processes are all less than in the same process and can be obtained if we compare Table 3 to Table 4.

### 3.4. Analysis and Summary

(1)  *The Precision of the Prediction Model Based on Time Series Data-Driven Depends on the Integrality and Sufficiency of History Data.* Machine learning algorithms, such as BPNN and SVM, are effective methods for utilizing history data to find the prediction model, but the model's precision is decided by the training data. If the data used for the model do not include all possible inputs and outputs, the model will not be suitable to describe the mapping relationship between them.

In Section 3.2, the simulation is during the same process of icing; it is obvious that the precision is satisfactory before the shelling phase and the error is less than 200 kg. However, in the shelling phase, that is unacceptable, especially in Figure 6, where the error is greater than 600 kg. This is dependent on the training data, which does not cover the shelling phase; so it is imprecise.

When compared with the results of Section 3.3, the simulation is performed during different stages in the icing process; consequently the error variances are less than those of Section 3.2 whether the prediction length is short or long. It is because of this the data for training input involves whole phases of the icing process, including inexistence, rising, and shelling (melting). As the shelling process is very important for diagnosing the safety of power transmission line [32], the integrality and sufficiency of history data is very necessary for prediction of it.

(2)* The Precision of Prediction Model Is Related to the Length of Prediction. If Prediction Length Were Short, the Prediction Error Would Be Small.* According to the information theory [33] that the correlativity would increase if the time distance decreases. This means the value of the icing load at time *n* is correlative to the value at time *n* − *k*, and the correlativity would decrease if *k* increased.

As the results of Tables 2–4 show, when *k* increases, the prediction error will also increase. If *k* < 5, the error variance will be less than 30% of max icing load. That means if the prediction length is less than two hours, it will be helpful for power grid departments to take actions in advance for potential icing disasters along the Tao-Luo-Xiong Line. But if the length of prediction is more than two hours, the method about deep learning [34] maybe is a good choice.

(3)* The Precision of the Prediction Model Based on a Data-Driven, Multivariable Time Series Is Better Than One Based on a Single Variable Time Series.* According to Takens' embedding principle [23], if time delay and embedding dimensions are suitable, the results of a prediction based on a single variable time series would be satisfactory. However, there exist some uncertainty and immaturity in the single variable time series; so we cannot confirm that the phase space adequately describes the process of a complex dynamics system. Multivariable time series are comprised of more dynamic information than a single variable.

As the analysis of Section 2.1 shows, the icing process along the transmission line is complex and decided by multimeteorological factors. Therefore, if only based on a single variable time series, such as the ARIMA model, it could not describe the icing process exactly. The results of Tables 2–4 also indicate the analysis above.

## 4. Conclusions

The transmission line icing is a nonlinear and high dimension process. The advantage of using an automatic monitoring system for power transmission lines is that a large amount of process data is available to diagnose. Because complex line icing processes cannot be easily represented using accurate mathematical models, a data-driven method must be considered. After analyzing the Makkonen model of the icing process, we determined that the line icing is a multivariate time series process.

Consequently, in this paper a model based on multivariable time series has been presented to predict the icing load of the transmission line. In this model, time effects of micrometeorology parameters for the icing process were analyzed, and the phase-space reconstruction theory and machine learning methods were applied, which utilized the historic multivariable time series data to establish the model. As the characteristic of fitfulness in line icing, the simulations were carried out during the same icing process or different process for testing the precision and robustness of the model.

According to the results of simulation for the Tao-Luo-Xiong Transmission Line, this model has a good accuracy of prediction in different processes if the prediction length is less than five. That means the maximal prediction time is approximately two hours, which would be helpful for power grid departments when deciding to take action in advance to address potential icing disasters.

## Figures and Tables

**Table 1:** Delay time *τ*_*i*_ and embedding dimension *d*_*i*_.

Various	*q*_*w*_	*T*_*P*_	*H*	*W*_*S*_	*W*_*D*_	*A*_*p*_	*S*_*L*_
*τ*_*i*_	2	0	0	0	0	0	0
*d*_*i*_	5	1	1	1	1	1	1

**Table 2:** The error variances of icing load prediction in same icing process for the first serious icing process from (12/17/2009~12/20/2009).

Prediction length *k*	1	2	3	4	5	6
Standard deviation (multivariable prediction based on BPNN)	108.6	134.5	142.8	181.7	255.6	297.5

Standard deviation (single variable prediction based on ARIMA)	151.9	194.7	263.5	289.3	320.1	386.2

**Table 3:** The error variances of icing load prediction in same icing process for the second serious icing process (1/10/2010~1/12/2010).

Prediction length *k*	1	2	3	4	5	6
Standard deviation (multivariable prediction based on BPNN)	136.1	149.4	172.5	210.6	282.1	313.6

Standard deviation (single variable prediction based on ARIMA)	145.2	204.9	288.7	293.6	368.4	407.4

**Table 4:** The error variances of icing load prediction in different icing process.

Prediction length *k*	1	2	3	4	5	6
Standard deviation (multivariable prediction based on BPNN)	71.2	93.5	135.8	178.9	266.6	324.7

Standard deviation (single variable prediction based on ARIMA)	126.3	133.3	171.1	229.9	317.2	356.8
